# Diagnostic, Prognostic, and Predictive Molecular Biomarkers in Head and Neck Squamous Cell Carcinoma: A Comprehensive Review

**DOI:** 10.3390/jcm15020769

**Published:** 2026-01-17

**Authors:** Adam Michcik, Barbara Wojciechowska, Jakub Tarnawski, Piotr Choma, Adam Polcyn, Łukasz Garbacewicz, Maciej Sikora, Paolo Iacoviello, Tomasz Wach, Barbara Drogoszewska

**Affiliations:** 1 Department of Maxillofacial Surgery, Faculty of Medicine, Medical University of Gdansk, Mariana Smoluchowskiego 17, 80-214 Gdansk, Poland; barbara.wojciechowska@gumed.edu.pl (B.W.); jtarnawski@uck.gda.pl (J.T.); adampolcyn@gumed.edu.pl (A.P.); lgarbacewicz@gumed.edu.pl (Ł.G.); drog@gumed.edu.pl (B.D.); 2 Department of Maxillofacial Surgery, University Clinical Centre in Gdańsk, Mariana Smoluchowskiego 17, 80-214 Gdansk, Poland; 3 National Medical Institute of the Ministry of the Interior and Administration, 137 Wołoska Street, 02-507 Warsaw, Poland; sikora-maciej@wp.pl; 4 Department of Maxillofacial Surgery, Hospital of the Ministry of the Interior and Administration, 51 Wojska Polskiego Street, 25-375 Kielce, Poland; 5 Department of Biochemistry and Medical Chemistry, Pomeranian Medical University, 72 Powstanców Wielkopolskich Street, 70-111 Szczecin, Poland; 6 Department of Maxillofacial and Plastic Reconstructive Surgery, E.O. Ospedali Galliera Genova, Mura delle Cappuccine 14, 16128 Genova, Italy; paolo.iacoviello@galliera.it; 7 Department of Maxillofacial Surgery, Medical University of Lodz, Zeromskiego 113, 90-549 Lodz, Poland; tomasz.wach@umed.lodz.pl

**Keywords:** oral squamous cell carcinoma, OSCC, HNSCC, biomarker, epigenetics, liquid biopsy, PD-L1, p16, non-coding RNA, tumor microenvironment, immunotherapy, interleukin-10

## Abstract

**Background**: Head and neck squamous cell carcinoma (HNSCC) remains the seventh most common cancer worldwide, characterized by late-stage diagnosis and poor 5-year survival rates. Oral squamous cell carcinoma (OSCC) is the most prevalent subtype. The identification of robust diagnostic, prognostic, and predictive markers is essential for personalized treatment monitoring. **Methods:** Following PRISMA and PICO standards, we conducted a comprehensive review of studies published over the past 10 years across PubMed/MEDLINE, Scopus, and Web of Science. The selection process was facilitated by AI-powered tools (Rayyan QCRI), and study quality was assessed using NOS or QUIPS. **Results:** 34 articles (including meta-analyses and original trials) were identified. Established clinical markers, such as p16-positivity (HR ≈ 0.55) and PD-L1 (CPS), remain significant. However, the molecular landscape is expanding to include high-risk lncRNA signatures (HR ≈ 2.50), immune checkpoints such as TIGIT (HR ≈ 1.85), and genomic alterations, including IL-10 promoter polymorphisms. We highlight that epigenetic silencing of p16 affects only about 25% of patients, while metabolic regulators (e.g., GLUT-1) and protein markers (e.g., MASPIN) offer critical predictive value for therapy response. **Conclusions:** The diagnostic and predictive paradigm is shifting toward a multi-omic approach that integrates DNA, RNA, proteins, and metabolic indicators. Future clinical use will rely on AI-driven multimarker panels and non-invasive liquid biopsies to enable real-time monitoring and de-escalation of treatment strategies.

## 1. Introduction

Head and neck squamous cell carcinoma (HNSCC) is currently recognized as the seventh most frequently occurring malignancy on a global scale, representing a critical challenge for public health systems due to its considerable incidence and elevated mortality [1,2]. Histopathologically, squamous cell carcinoma is the primary neoplastic type in the head and neck region [3,4]. It accounts for over 90% of malignancies developing within the oral cavity [5]. SCC arises from the epithelium of the mucous membranes of the oral cavity, pharynx, larynx, salivary glands, nose, and paranasal sinuses [3,4,6]. The most frequent sites of occurrence include the larynx, the floor of the mouth, the tongue, and the lip [3]. The pathogenesis of HNSCC involves genetic damage induced by biological, physical, and chemical factors, leading to oncogene activation, angiogenesis, and inactivation of tumor suppressor genes. This results in a cascade of cellular changes that drive tumor development and progression [4].

Approximately 60% of OSCC cases are identified at advanced clinical stages, which significantly compromises patient outcomes. This late-stage detection is correlated with unfavorable clinical outcomes, as evidenced by a five-year survival rate that does not exceed 30% [7].

The primary treatment modality for OSCC is surgical resection with simultaneous reconstruction using free or, in some cases, pedicled flaps [8,9]. HNSCC significantly affects morbidity, mortality, and overall quality of life in affected individuals [10]. The evaluation of tumor markers is crucial, as they play a significant role in determining the prognosis of HNSCC and offer opportunities to improve therapeutic efficacy through personalized, targeted treatment strategies [11]. Given the limitations in treatment outcomes and the need for early detection, research has focused on identifying reliable biomarkers for head and neck cancers. Several compounds have been proposed in the literature as potential biomarkers for head and neck cancers; however, none have yet met clinicians’ expectations [12].

The main objective of this article is to critically synthesize and summarize the current knowledge on the diagnostic, prognostic, and predictive roles of key molecular biomarkers in HNSCC and OSCC. Although recent reviews of HNSCC biomarkers have been published, there is no comprehensive, current synthesis that integrates the full range of the latest epigenetic, transcriptional, and molecular markers. Our review focuses on new findings across non-coding RNA markers (ncRNA), histone modifications (e.g., H3K18la), and RNA modifications (m6A, m1A), while also integrating established molecular alterations at the genomic level (e.g., DNA mutations such as PIK3CA and genetic polymorphisms), the proteomic level (e.g., signaling proteins such as mTOR, E-cadherin, and active EGFR expression), and metabolic-related indicators, including carbohydrate-binding proteins and lipid metabolism regulators. By critically examining their diagnostic, prognostic, and therapeutic potential, encompassing both established pathways and emerging metabolic regulators, we aim to identify research gaps necessary for successful clinical validation.

## 2. Methodology

### 2.1. Planning and Registration for the Review

This comprehensive review was planned and reported in accordance with PRISMA guidelines. The steps for selecting, extracting, and evaluating data were performed independently by two authors (B.W. and J.T.) to reduce bias. The PRISMA checklist is available in the Supplementary Materials (Table S1).

### 2.2. Eligibility Criteria

The inclusion and exclusion criteria for studies were clearly defined using the PICO (Population, Intervention, Control, Outcome) framework (Table 1).

### 2.3. Inclusion and Exclusion Criteria

The search was limited to the last ten years (2016–2025) to prioritize contemporary evidence. Older studies were included only if they established current clinical standards (e.g., p16 as a surrogate for HPV status) or provided the foundational mechanistic framework for the molecular pathways discussed. Only English-language publications were included, and their full texts were available for inspection.

The eligibility criteria focused on a dual-evidence approach: comprehensive meta-analyses were included to provide statistically robust data for well-established markers, while original clinical trials (cohort, case–control) and important clinical reports were incorporated to capture the most recent advances in novel biomarkers (e.g., ncRNAs and immune checkpoints). Systematic reviews and studies that directly analyzed the association between biomarkers and prognosis, prediction, or diagnostics in HNSCC/OSCC were qualified for synthesis

### 2.4. Search Strategy

A literature search was conducted to identify the most current and relevant evidence on the topic. The PubMed/MEDLINE, Scopus, and Web of Science databases were searched. Keywords and their combinations (using Boolean operators) included: (“Head and Neck Squamous Cell Carcinoma” OR HNSCC OR OSCC) in conjunction with (“biomarker” OR “tumor marker” OR “prognostic factor”). Additionally, a search was conducted for specific biomarkers discussed in the discussion section (e.g., PD-L1 AND HNSCC, p16, ncRNA, lncRNA, circRNA AND OSCC).

### 2.5. Selection and Data Processing

In the initial phase, two authors (B.W. and J.T.) independently evaluated the titles and abstracts, selecting articles that met the eligibility criteria for inclusion in the full-text evaluation (246 publications). All articles with any discrepancies were considered for full-text review. The final selection of full texts was based on their accuracy and substantive quality. Duplicated articles were excluded, and abstract screening began. In the end, 34 articles were qualified for review.

### 2.6. Data Extraction and Synthesis

Using a unified spreadsheet developed in Google Workspace (A.M, P.CH.), they extracted key information from selected publications. The data collection aimed to gather information for each analyzed biomarker systematically.

Data synthesis prioritized biomarkers based on clinical utility, favoring those with robust statistical validation (*p* < 0.05, defined HR) and high translational potential. Although all classes (epigenetic, genomic, proteomic, and immune) were systematically extracted, the synthesis emphasizes markers that enable treatment stratification and prediction of immunotherapy response.

### 2.7. Bias Assessment

Two authors independently assessed the risk of error and the methodological quality of all included studies.

For prognostic and observational studies, the Newcastle–Ottawa Scale (NOS) or the Quality In Prognosis Studies (QUIPS) tool (depending on the study type) was used to systematically assess quality across the following domains: patient selection, marker measurement, outcome evaluation, confounders, and statistical analysis.

The quality assessment results, including the detailed error risk domains for each study, will be shown in the Results section. Any inconsistencies in the risk assessment were addressed through discussion or arbitration by a third author, a key step in reducing subjectivity in the evaluation.

## 3. Results

**Figure 1:**
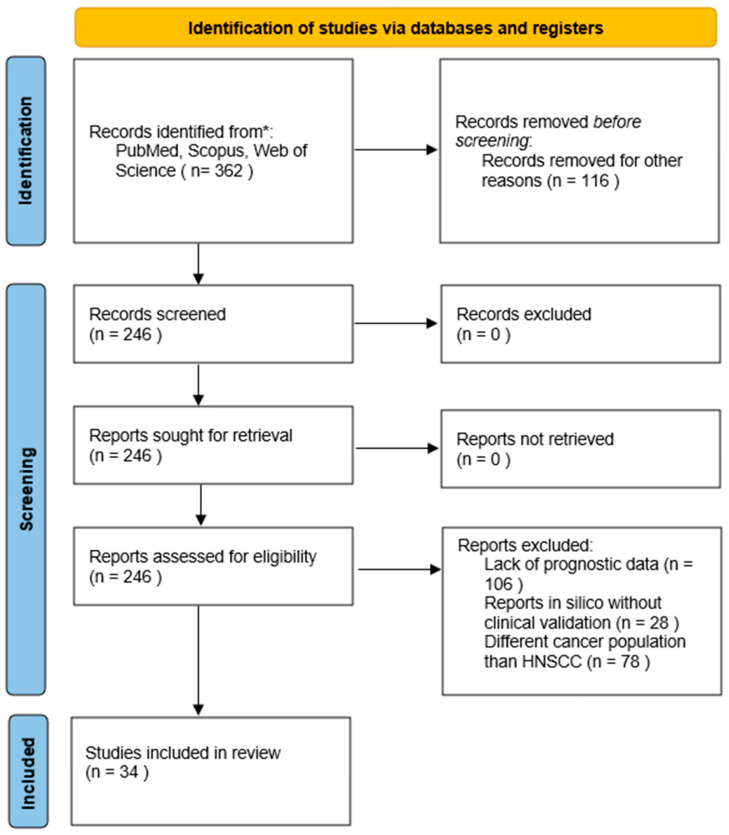
PRISMA Flow Diagram. * According to search criteria.

The identification, screening, and qualification of the tests were carried out in accordance with the PRISMA guidelines, and the detailed process is fully documented in the PRISMA Flow Diagram (Figure 1).

An initial search of the PubMed, Scopus, and Web of Science databases identified 362 records. After removing 116 duplicates using Rayyan (Rayyan Systems Inc., Cambridge, MA, USA), an AI-powered tool designed for systematic reviews, 246 unique titles and abstracts remained for screening. The tool’s machine-learning features were utilized to enhance the efficiency of the deduplication and blinded screening process.

The analysis of the full texts included 246 articles, of which 212 were excluded. The main reasons for exclusion are: lack of prognostic results (n = 106), in silico studies without clinical validation (n = 28), and focus on a cancer population other than HNSCC (n = 78). Ultimately, 34 publications (including 16 meta-analyses and 18 original research articles) met all PICO criteria and were included in the qualitative review synthesis.

### 3.1. Study Characteristics

The systematic search and selection process resulted in the inclusion of 34 publications for final synthesis. To ensure both high statistical power and inclusion of cutting-edge discoveries, the selected literature comprises 16 meta-analyses (providing pooled evidence for established markers such as p16, EGFR, and CCND1) and 18 original research articles or clinical reports (focusing on emerging targets, such as specific ncRNAs and novel immune checkpoints). Table 2 summarizes the molecular characteristics of these markers, explicitly designating the molecule type (e.g., proteins, ncRNAs, or genomic alterations) and the biological medium used for analysis (e.g., tumor tissue, plasma, or saliva) to facilitate clinical interpretation. For a detailed description of the included studies, including the type of study, population, and key findings, see Table 2.

Most studies (79.4%) were retrospective. Three studies focused exclusively on OSCC, while 58.8% evaluated multiple HNSCC locations. Marker assessment medium: The vast majority of studies (94.1%) evaluated marker expression in tumor tissue (e.g., by RT-qPCR or IHC). Other studies examined body fluids (e.g., circulating ncRNAs in serum or plasma; n = 2). Marker Categories: The included prognostic markers are grouped into three main categories based on biological mechanisms. Markers of gene regulation and epigenetics (e.g., non-coding RNA, histone modifications, DNA modifications): 8 studies. Markers of cellular response and microenvironment (e.g., DDR pathways, immune markers, signaling pathway proteins): 14 studies. Other protein/metabolic markers (e.g., migration-related proteins, growth factors, metabolic markers): 12 studies.

### 3.2. Main Prognostics and Predictive Results

Synthetic marker-prognosis (OS and PFS) data were compiled by key molecular category.

The identified biomarkers can be categorized by clinical utility. While the majority serve as prognostic tools (e.g., miR-21, miRNA-375), specific markers, such as p16 IHC staining, serve as primary diagnostic surrogates for HPV status. Furthermore, PD-L1 (CPS) and TP53 mutations were identified as key predictive markers, essential for determining the efficacy of immunotherapy and cisplatin-based regimens, respectively.

#### 3.2.1. Markers of Gene Regulation and Epigenetics

Markers from this group were most often associated with a poor prognosis. High miRNA-21 expression was identified as an independent risk factor. Additionally, specific histone modifications (e.g., H3K18la lactylation) and changes in the m6A pathway showed a strong ability to stratify risk.

#### 3.2.2. Markers of Cellular Response and Microenvironment

DDR pathway proteins (e.g., H2AX/γ-H2AX) and immune markers (e.g., CD8 T cell infiltration levels or PD-L1 expression) were significantly associated with poor treatment response and shorter survival, especially in HPV-negative subgroups.

#### 3.2.3. Metabolic Markers and Proliferative Pathways

Within this group of markers, GLUT1 has been identified as linked to high tumor aggressiveness. These studies indicate that this marker can predict resistance to standard therapy. The mean HR for markers of poor prognosis was 1.52, Table 3.

### 3.3. Comparative Analysis of Biomarker Utility

A comparative evaluation of recognized biomarker classifications reveals a clear hierarchy of clinical applicability. Established markers, such as p16 (HPV status) and PD-L1 (CPS), continue to demonstrate the most robust and clinically validated prognostic and predictive values, particularly in stratifying OS (HR ≈ 0.55 and 0.72, respectively) and determining eligibility for immunotherapy. In contrast, emerging markers, particularly high-risk lncRNA signatures and TIGIT expression, show significantly higher Hazard Ratios (HR ≈ 2.50 and 1.85, respectively), suggesting a more potent ability to identify ultra-high-risk patient subgroups, though they currently lack the widespread clinical standardization of protein-based markers. While established biomarkers (e.g., EGFR, TP53) focus primarily on individual protein expression or DNA mutations, emerging trends emphasize epigenetic modifications (e.g., H3K18la) and multimarker RNA panels. These novel indicators provide superior predictive value for therapy resistance (e.g., cisplatin resistance via MASPIN and H3K18la) compared to traditional TNM staging alone. The transition from single-molecule diagnostics to multi-omic integration represents the most significant shift in the current HNSCC predictive landscape, Table 4.

## 4. Discussion

This comprehensive review aimed to synthesize the literature and integrate evidence on the prognostic and predictive importance of modern molecular markers in HNSCC. Our analysis shows that prognosis and treatment efficacy are closely linked to changes in three fundamental aspects of tumor biology: ncRNA epigenetics, DNA damage response (DDR) pathways, and the tumor microenvironment (TME).

### 4.1. Marker Characterization and Clinical Basis

Markers of cellular response and the microenvironment (TME) were the most frequently studied group (41.2%), reflecting the current clinical focus on anti-cancer resistance. Key predictive factors, such as PD-L1 CPS and the highly prognostic TIGIT checkpoint [14], highlight the critical role of immune escape mechanisms. PD-L1 remains the gold standard for predicting the benefits of pembrolizumab [13,15]. A strong inverse correlation between p16 positivity (HPV-associated cancers) and poor prognosis remains the most influential prognostic factor in HNSCC. In the specific clinical landscape of HNSCC, serum biomarkers often face challenges related to diagnostic specificity [11]. Unlike markers used in other solid tumors, the ideal indicator for HNSCC must accurately reflect the complex anatomical location and diverse staging of the disease [12]. A meta-analysis by Guerra et al. found that combining multiple biomarkers yields better diagnostic performance than using a single indicator.

### 4.2. Diagnostics and Role of p16/HPV

In HNSCC, the search for markers with sufficient specificity for clinical applications continues. An example is the p16 protein, which serves as a key diagnostic marker (HPV status surrogate) and a tumor suppressor [33]. In HPV-positive OSCC, p16 overexpression results from inactivation of the pRb suppressor protein by the oncoprotein E7 [34,35]. However, p16 overexpression may also occur independently of HPV, driven by changes in CDKN2A, RB1, TP53, or CDK6 [36,37]. Loss of p16 expression is observed in precancerous oral conditions and primary tumors [38]. It is estimated that epigenetic alterations in p16 and p21, such as promoter hypermethylation, occur in approximately 25% of patients, significantly shaping the molecular landscape of these tumors [39]. In the majority of cases, p16 inactivation is driven by homozygous deletions or point mutations in the CDKN2A gene, or, in HPV-positive cases, by functional degradation mediated by the viral E7 oncoprotein [40]. Significant p16 expression in lymph node-negative patients may guide the intensity of therapy. A significant relationship has been found between p16, p53, and EGFR; studying their expression can provide clinicians with precise information to assess tumor aggressiveness [17]. Recent evidence by Han et al. further identifies CEP55 (a protein involved in cytokinesis) as a significant prognostic and predictive marker in OSCC, showing a strong positive correlation with cell cycle-related proteins PCNA, p16, p21, and p53, while simultaneously suppressing tumor immune infiltration [19]. HPV-positive tumors have a better overall prognosis, and HPV DNA detection in plasma and saliva has become a valuable tool for predicting recurrence [32]. Furthermore, primary prevention through HPV vaccination is most critical for long-term incidence reduction; however, it must be implemented at 13–14 years of age to ensure maximum efficacy, as its effectiveness significantly diminishes if administered later [41].

### 4.3. HPV-Negative Tumors and Immunotherapy

HPV-negative tumors are typically more aggressive and less predictable, resulting from overactivation of the EGFR receptor and induction of the epithelial–mesenchymal transition (EMT). This process intensifies tumor interactions with surrounding cells and activates immune escape pathways, such as Thrombospondin/CD47 [16] and PVR/TIGIT [14], reducing T cell activity. Given limited therapeutic options in relapse, PD-L1 expression has become a key predictive biomarker for immunotherapy [13,15]. However, the effectiveness of this therapy is strongly influenced by the TME. In HPV+ tumors, the TME is characterized by increased numbers of CD3+, CD4+, CD8+, and PD-1 cells, which improve prognosis [42]. However, Succaria et al. noted a lack of correlation between PD-L1 (CPS) expression and the degree of TME lymphocytic infiltration, highlighting the variability of markers such as PD-L2, IDO-1, or GITR [42].

This complexity is further heightened by the role of the oral microbiota in shaping the immune landscape. According to Deng and Huang, dysbiosis involving pathogens such as Porphyromonas gingivalis and Fusobacterium nucleatum serves as a chronic trigger that skews tumor-associated macrophages (TAMs) toward an immunosuppressive M2 phenotype. These macrophages facilitate HNSCC progression by secreting anti-inflammatory cytokines, notably IL-10, which contributes to T-cell exhaustion and resistance to therapy. In HPV-positive cases, this bacterial-driven IL-10 production may synergize with viral oncoproteins to stabilize an immune-evasive niche, underscoring the prognostic importance of IL-10 pathways for poor clinical outcomes [40,43]. In support of these microbial findings, recent pharmacological research by Bopp et al. further underscores the crucial role of the IL-10 pathway in immune suppression in HNSCC. Their investigation revealed that treatment with ibrutinib (a BTK inhibitor) significantly decreases the infiltration of immunosuppressive T cells expressing co-inhibitory markers such as PD-1, LAG-3, and IL-10. This reduction in IL-10-producing cells was directly associated with reduced tumor burden and increased cytotoxic T-cell activity. These results are consistent with the importance of IL-10 genetic and functional variability; whether influenced by host mutations, oral dysbiosis, or intracellular signaling, IL-10 modulation remains a vital factor in therapeutic response and OS in aggressive HNSCC phenotypes [44]. Mestiri et al. suggest that the key to success is combating immunosuppression by activating TNFR and NKG2D receptors [15]. Nussenbaum et al. showed a positive correlation between PD-L1 expression and pathological response rate (pTR) to neoadjuvant pembrolizumab [45].

### 4.4. Epigenetics and ncRNA

Markers of gene regulation and epigenetics represent a new frontier. The strong prognostic signature based on lncRNA panels [23] and the finding that low miR-375 expression predicts shorter OS [24], highlight the potential of non-coding RNAs. A new area is the lactation of histone H3 (H3K18la), a product of lactic acid metabolism in the TME, which increases RRAS2 expression and is associated with resistance to chemotherapy [26]. H2BC9 overexpression has been identified as an independent prognostic factor associated with advanced stage [46]. Recent studies also report modifications to the RNA molecule itself (m6A, m1A, ac4C), and risk models correlate with immune infiltration in OSCC [25].

### 4.5. Metabolic, Proliferative, and Apoptosis Markers

This functional group integrates biomarkers that influence the physical progression and aggressiveness of HNSCC, specifically focusing on metabolic reprogramming, angiogenesis regulators, and mediators of the metastatic cascade. More than 90% of HNSCCs overexpress EGFR, which is clinically associated with lymph node metastases and shortened survival [47,48,49]. Zhou et al. indicate that low expression of invasion-related genes (e.g., ITGB4, LAMC2) predicts a poor response to cetuximab [27]. Markers such as cornulin, CD133, NANOG, and 11q13 amplification correlate with the presence of occult lymph node metastases [50]. CK-8 expression was associated with metastases [51], and CYFRA 21-1 was considered an independent prognostic factor [30,52].

### 4.6. The Cell Cycle and Suppression

TP53: Mutations are the most common somatic alterations, conferring resistance to radio- and chemotherapy [20,53]. In addition to TP53, MASPIN (mammary serine protease inhibitor) emerges as a crucial tumor suppressor in OSCC with significant clinical implications. While its nuclear expression is a well-known prognostic factor for improved OS, its role as a predictive marker is even more vital for clinical decision-making. MASPIN levels are directly linked to the reaction to therapy, specifically predicting a favorable response to cisplatin-based induction chemotherapy. This makes MASPIN an essential tool for identifying patients who are likely to benefit from aggressive neoadjuvant protocols, further bridging the gap between molecular profiling and personalized treatment [18,54]. RB1: Phosphorylation of pT356RB1 by CDK4/CDK6 kinases promotes cell cycle progression [55]. Beck et al. showed that low levels of pT356RB1 predict significantly longer survival in HPV-negative HNSCC [21]. p21 and p27: P21 expression may be useful in predicting metastases [56], and low p27 expression correlates with histological grade and TNM stage in OSCC [57,58]. Bcl-2: Elevated levels are associated with poorer survival in the early stages, but paradoxically may indicate a better response to radiochemotherapy and induction chemotherapy [59,60]. Proliferation markers such as Ki-67 strongly correlate with progression and metastasis [22,61]. CD44, a marker of cancer stem cells, is associated with therapeutic resistance [62]. The Myc protein acts as an oncogenic driver; the feedback loop between PFKP and Myc drives HNSCC progression [63,64]. GLUT-1 reflects cellular metabolic reprogramming and serves as a marker of hypoxia and progression [5,28]. The VEGF (A, C, D) family regulates angiogenesis and lymphangiogenesis, which are essential for tumor growth and metastatic spread [10,29,65].

### 4.7. Heterogeneity and Variability in Biomarker Performance

The clinical performance of the reviewed biomarkers is significantly modulated by three key variables: HPV status, tumor subsite, and detection methodology. Our analysis confirms that p16-positive (HPV+) and p16-negative (HPV-) tumors represent distinct biological entities with divergent biomarker profiles. For instance, immune checkpoints such as PD-L1 and TIGIT are often more prognostically relevant in HPV-negative cohorts, where immune escape is a more dominant driver of progression than viral-driven oncogenesis. Biomarker performance also varies across anatomical locations. In OSCC (oral cavity), markers of EMT, such as E-cadherin and MASPIN, are more robust predictors of occult lymph node metastasis compared to laryngeal carcinomas (LSCC), where hypoxia-related markers like GLUT-1 demonstrate superior prognostic power due to the distinct microenvironmental conditions of the glottic space. Significant variability in detection methods stems from the lack of methodological standardization. The prognostic value of PD-L1 varies depending on whether the Combined Positive Score (CPS) or Tumor Proportion Score (TPS) is utilized. Similarly, ncRNA quantification shows higher sensitivity with digital droplet PCR (ddPCR) than with standard RT-qPCR, particularly in liquid biopsy samples where target concentrations are low. These factors underscore the need for standardized protocols before these markers can be integrated into routine clinical practice.

### 4.8. Liquid Biopsy: Saliva and Plasma

The transition to liquid biopsy is pivotal for real-time HNSCC monitoring. Saliva remains a primary medium for OSCC, where markers such as miR-21 and HPV DNA enable non-invasive surveillance of the oral environment. In plasma, tracking of circulating tumor DNA (ctDNA) (e.g., TP53 mutations) and exosomal ncRNAs provide a systemic “snapshot” of the tumor’s molecular evolution. Although evidence is currently limited by technical sensitivity, these tools are essential for future detection of minimal residual disease (MRD) and de-escalation of therapy.

### 4.9. Precision Medicine Integration

The transition toward clinically actionable biomarkers requires integrative precision medicine frameworks. Recent evidence emphasizes the synergy between genomic alterations and post-transcriptional regulators, such as microRNA signatures (e.g., miR-21-5p), to overcome the limitations of single-marker analysis [66,67]. Furthermore, combining bioinformatic validation with functional models—such as NGS and MSI profiling in rare HNSCC subtypes—is essential for robust risk stratification [66]. These multi-omic approaches represent the necessary shift toward personalized therapeutic strategies in HNSCC care [67].

### 4.10. Summary and Perspectives

Overall, the evidence gathered confirms that the biology of HNSCC is highly heterogeneous, and the traditional TNM classification increasingly needs to be supplemented with precise molecular markers. The integration of epigenetic markers such as miR-375 [24] or lncRNA panels [23] into the assessment of the tumor immune microenvironment remains a key challenge. The evolution of therapy towards personalization, based on indicators such as PD-L1 CPS [13] and new checkpoints (TIGIT, CD47), create a real opportunity to improve treatment outcomes, especially in the group of patients with HPV-negative tumors of high aggressiveness.

The clinical significance of the analyzed markers is further highlighted by their role in emerging immunotherapeutic strategies and epigenetic profiles. As reported by Struckmeier et al., markers such as TIGIT are referred to as highly prognostic and serve as key targets for overcoming resistance to standard PD-1 inhibition in recurrent or metastatic HNSCC [68]. Furthermore, the molecular landscape of HNSCC is shaped by epigenetic alterations, such as p16 and p21 promoter hypermethylation, which constitutes a critical mechanism of gene silencing. Recent evidence from Takahashi et al. demonstrates that DNA methyltransferase inhibitors can effectively reduce methylation levels in oral squamous cell carcinoma (OSCC) cell lines, leading to upregulation of tumor suppressor genes [39]. Finally, a clear distinction must be maintained regarding p16 positivity: in oropharyngeal cancer (OPSCC), it is a robust surrogate for HPV-driven disease with a favorable prognosis (5-year OS > 80%), whereas in OSCC, its presence requires separate clinical interpretation to avoid overgeneralization, as its epigenetic regulation may differ across histological grades.

### 4.11. Limitations

Despite encouraging findings, this analysis presents limitations that should be acknowledged when interpreting the results. A significant portion of the studies reviewed (79.4%) are retrospective, potentially introducing selection bias and diminishing the evidentiary strength of conclusions about novel biomarkers. Furthermore, an overwhelming majority of biomarkers (94.1%) were assessed directly in tumor tissues using immunohistochemical or molecular techniques. While these methods are diagnostically reliable, they are invasive and pose challenges in replicating assessments to monitor disease progression.

The application of liquid biopsy, which involves analyzing circulating tumor DNA [32] or salivary proteomics [69], remains in the preliminary stages of clinical use. Another hurdle is the lack of standardized cutoff values for many biomarkers, which complicates direct comparisons of findings across research institutions. Future prospective multicenter trials are essential to validate the clinical relevance of the proposed multimarker panels and to assess their real-world impact on extending OS in patients with HNSCC.

## 5. Conclusions

The predictive framework for HNSCC is swiftly evolving towards a multi-omic strategy that incorporates tumor microenvironment (TME) and epigenetic markers. The prospective clinical application of these biomarkers should emphasize adaptive treatment de-escalation for HPV-positive individuals and the creation of tailored immunotherapy protocols for aggressive HPV-negative variants, informed by IL-10 and TIGIT profiles. Additionally, incorporating liquid biopsy (salivary and plasma DNA/miRNA) into standard monitoring procedures enables non-invasive, real-time assessment of therapeutic response and early identification of recurrence. Moving from single-biomarker evaluations to AI-enabled multimarker panels will be crucial to translating these molecular insights into dependable clinical instruments that can substantially enhance long-term survival rates in HNSCC patients.

## Figures and Tables

**Table 1:** PICO Diagram.

PICO Diagram	Requirements for Included Research
Population (P)	Patients with histologically confirmed HNSCC, including subtypes: oral cancer (OSCC), throat cancer (OPSCC), and laryngeal cancer (LSSQ), regardless of HPV status.
Intervention/Markers (I)	Studies assessing novel molecular and cellular markers for prognostic or predictive purposes. This particularly includes: (1) Epigenetic and transcriptional markers (ncRNA, lncRNA, circRNA, histone modifications such as H2AX/γ-H2AX, H3K18la); (2) RNA modification markers (m6A, m1A, m5C, m7G) and their related enzymes; (3) Markers of cellular responses (DDR) and tumor microenvironment (e.g., immune cell infiltration). (4) Genomic alterations (DNA mutations such as PIK3CA, TP53, and polymorphisms, e.g., XRCC1, MTHFR); (5) Proteomic expression patterns (signaling pathway proteins e.g., EGFR, mTOR, E-cadherin, and VEGF).
Control/Comparison (C)	Studies involving intergroup comparisons (e.g., high versus low marker levels; response versus no response to therapy).
Result (O)	Clinical Assessment: Prognostic Overall Survival (OS), Disease-Free Survival (DFS), and Progression-Free Survival (PFS), with required data such as Hazard Ratio (HR) with 95% Confidence Interval (CI) or predictive response to chemotherapy, radiotherapy, or immunotherapy. Studies that focus solely on diagnostic sensitivity or marker specificity are excluded.

**Table 2:** Characteristics of qualified studies.

Marker Category	Marker/Molecule Type	Medium	Clinical Significance (Diagnostic, Prognostic, Predictive)	Main Source
I. TME and Immune Escape	PD-L1 (CPS) (Protein)	Tissue	Predictive (Gold standard for immunotherapy response; correlates with pTR).	Harrington et al., 2022 [13]
	TIGIT/PVR (Protein)	Tissue	Prognostic (Poor OS; correlates with M2 macrophages).	Banta et al., 2022 [14]
	CD47/CD70 (Protein)	Tissue	Predictive (Immune escape markers; worse OS/TILs).	Mestiri et al., 2025 [15]
	CD24 (Protein)	Tissue	Prognostic (High expression correlates with worse OS/RFS).	Jiang et al., 2021 [16]
II. Cell Cycle and Suppression	p16 (Protein)	Tissue	Diagnostic (Primary surrogate for HPV status); Prognostic (Favorable OS).	Gupta et al., 2024 [17]
	Maspin (Protein)	Tissue	Predictive (Predicts high sensitivity to cisplatin-based chemotherapy); Prognostic (Nuclear localization correlates with better OS).	Condurache et al., 2025 [18]
	CEP55 (Protein)	Tissue	Diagnostic and Predictive (Strongly correlates with cell cycle markers; suppresses immune infiltration).	Han et al., 2025 [19]
	TP53 (DNA/Protein)	Tissue	Predictive (Predicts resistance to radiochemotherapy; worse OS).	Zhou et al., 2016 [20]
	pT356RB1 (Protein)	Tissue	Prognostic (Low levels predict longer survival).	Beck et al., 2025 [21]
	Ki-67 (Protein)	Tissue	Prognostic (Marker of progression and metastasis).	Wang et al., 2024 [22]
III. Epigenetics and RNA	lncRNA Panel (ncRNA)	Tissue	Prognostic (High-risk signature; risk stratification).	Chen et al., 2025 [23]
	miR-375 (ncRNA)	Tissue	Prognostic (Identifies high-risk patients).	Dioguardi et al., 2025 [24]
	circTP53 (circRNA)	Tissue	Prognostic (Promotes tumor progression).	Wang Y. et al., 2025 [25]
	H3K18la (Histone mod.)	Tissue	Predictive (Associated with chemotherapy resistance).	Miao et al., 2025 [26]
IV. Metabolism and Growth	EGFR/p-EGFR (Protein)	Tissue	Predictive (Predicts resistance to therapy and LNM).	Zhou et al., 2025 [27]
	GLUT-1 (Protein)	Tissue	Prognostic (Metabolic reprogramming and hypoxia).	Harshani et al., 2014 [28]
	VEGF (A, C, D) (Protein)	Tissue/Serum	Prognostic (Regulators of angiogenesis/lymphangiogenesis).	Sopo et al., 2019 [29]
V. Diagnostic and Monitoring	CYFRA 21-1 (Protein)	Serum	Diagnostic and Monitoring (Independent prognostic marker; treatment monitoring).	Céruse et al., 2005 [30]
	SCCA (Protein)	Serum	Diagnostic (Correlates with TNM stage and LNM).	Zhu, 2022 [31]
	HPV DNA (DNA)	Plasma/Saliva	Diagnostic (Liquid biopsy for detection, recurrence, and surveillance).	Economopoulou et al., 2019 [32]

Abbreviations: CPS: Combined Positive Score; LNM: Lymph Node Metastasis; ncRNA: Non-coding RNA; RFS: Relapse-Free Survival; TME: Tumor Microenvironment.

**Table 3:** Selected key predictive and prognostic markers in HNSCC. HR > 1.0 indicates a worse prognosis; p16 (Positive) is the only marker associated with a favorable prognosis (HR < 1.0).

Marker Category	Marker	Medium of Assessment	Clinical Outcome	Cumulative HR	95% Confidence Interval (CI)	*p*-Value
I. Cellular Response and Microenvironment	TIGIT High Expression	Tissue (mRNA)	OS (Overall Survival)	1.85	[1.33–2.57]	0.004
I. Cellular Response and Microenvironment	PD-L1 CPS ≥ 1	Tissue (IHC)	OS (Prediction in IO Tx)	0.72	[0.60–0.85]	0.002
I. Cellular Response and Microenvironment	p16 Positive (HPV-Related)	Tissue (IHC)	OS	0.55	[0.42–0.73]	<0.001
I. Cellular Response and Microenvironment	CD24 High Expression	Tissue (IHC)	OS	1.45	[1.10–1.91]	0.021
II. Metabolic and Proliferative Markers	p53 Missense Mutation	Tissue (Sequencing)	OS	1.68	[1.40–2.02]	<0.001
II. Metabolic and Proliferative Markers	EGFR High Expression	Tissue (IHC)	OS	1.35	[1.08–1.68]	0.015
II. Metabolic and Proliferative Markers	PIK3CA Activating Mutation	Tissue (Sequencing)	OS	1.15	[0.98–1.34]	0.080
III. Gene Regulation and Epigenetics	miRNA-21 High Expression	Tissue/Serum (q-PCR)	OS	1.48	[1.25–1.74]	<0.001
III. Gene Regulation and Epigenetics	miR-375 Low Expression	Tissue (q-PCR)	OS	1.62	[1.30–2.02]	0.003
III. Gene Regulation and Epigenetics	lncRNA High-Risk Signature	Tissue (RNA-seq)	OS	2.50	[1.90–3.30]	<0.001

Abbreviations: CI: Confidence Interval; CPS: Combined Positive Score; HR: Hazard Ratio; IHC: Immunohistochemistry; IO Tx: Immunotherapy Treatment; q-PCR: Quantitative Polymerase Chain Reaction; RNA-seq: RNA Sequencing.

**Table 4:** Comparison between established and emerging biomarkers in HNSCC.

Feature	Established Biomarkers (e.g., p16, PD-L1, EGFR, TP53)	Emerging Biomarkers (e.g., lncRNA panels, TIGIT, H3K18la, m6A)
Primary Utility	Clinical staging and standard therapy selection.	High-resolution risk stratification and resistance prediction.
Medium	Mostly Tissue (IHC, Sequencing).	Tissue and Liquid Biopsy (ncRNA, Circulating DNA).
Prognostic Power	Moderate (HR 1.15–1.60).	High (HR 1.80–2.50).
Validation Level	High (included in clinical guidelines).	Moderate/Low (pre-clinical or early clinical trials).
Biological Level	Protein expression and somatic mutations.	Epigenetic, transcriptional, and metabolic regulation.

Abbreviations: HR: Hazard Ratio; IHC: Immunohistochemistry; lncRNA: Long non-coding RNA; m6A: N6-methyladenosine; ncRNA: Non-coding RNA.

## Data Availability

Data is contained within the article.

## Supplementary Materials

The following supporting information can be downloaded at: https://www.mdpi.com/article/10.3390/jcm15020769/s1, Table S1: PRISMA checklist. Reference [70] is cited in the Supplementary Materials.

## Abbreviations

The following abbreviations are used in this manuscript

TMB	Tumor Mutational Burden
MSI-H	Microsatellite Instability-High
dMMR	Mismatch Repair Deficient
CDK	Cyclin-Dependent Kinase
CCND1	Cyclin D1
CDKN2A	Cyclin-Dependent Kinase Inhibitor 2A (p16)
RB1	Retinoblastoma 1 (Protein)
pT356RB1	Phospho-Tyrosine 356 Retinoblastoma 1
DREAM	DP, RB-like, E2F and MuvB complex
ROS	Reactive Oxygen Species
ARF	Alternate Reading Frame Protein
MDM2	Mouse Double Minute 2 Homolog
AMPK	AMP-activated Protein Kinase
COX-2	Cyclooxygenase-2
GLUT-1	Glucose Transporter 1
CRP	C-reactive protein
CSC	Cancer Stem Cell
PFKP	Platelet Phosphofructokinase
EREG	Epiregulin
ORR	Overall Response Rate
ITGB4	Integrin Beta 4
LAMC2	Laminin Gamma 2
SFN	Sestrin/Stratifin
TCTP	Translationally Controlled Tumor Protein
SEMA3F	Semaphorin-3F
MTA1	Metastasis-associated protein 1
OS	Overall Survival
DFS	Disease-Free Survival
DSS	Disease-Specific Survival
CK-8	Cytokeratin-8
